# Markers of Bone Metabolism in Patients With Chronic Pancreatitis and Pancreatic Ductal Adenocarcinoma

**DOI:** 10.1097/MD.0000000000001754

**Published:** 2015-10-23

**Authors:** Raffaele Pezzilli, Gian Vico Melzi d’Eril, Alessandra Barassi

**Affiliations:** From the Pancreas Unit, Department of Digestive System, Sant’Orsola-Malpighi Hospital, Bologna, Italy (RP); and Department of Health Sciences, University of Milan, Milan, Italy Bologna, Italy (ME, AB).

## Abstract

There are no studies comparing some of the most important markers, such as vitamin D, parathormone, osteocalcin, bone alkaline phosphatase, and calcium, in patients with chronic benign and malignant pancreatic diseases. Our objective was to comparatively evaluate serum markers of bone metabolism in patients with chronic pancreatitis and in those with ductal pancreatic adenocarcinoma. Sixty-three consecutive subjects were studied: 30 patients with a firm diagnosis of chronic pancreatitis and 33 having histologically confirmed pancreatic adenocarcinoma. Serum 25-hydroxyvitamin D, bone alkaline phosphatase, osteocalcin, parathormone, and calcium were determined using commercially available kits. Taking into consideration the clinical variables of all 63 patients studied, 25-hydroxyvitamin D was inversely correlated with only the body mass index (*P* = 0.007), whereas it was not correlated with age (*P* = 0.583) or fecal elastase-1 concentrations (*P* = 0.556). Regarding the other substances studied, parathormone was positively correlated with only the age of the patients (*P* = 0.015). Of the 5 substances studied, only bone alkaline phosphates were significantly different (*P* < 0.001) between patients with chronic pancreatitis and those with pancreatic ductal adenocarcinoma. Within the 2 groups of patients, the 23 patients with chronic pancreatitis without diabetes mellitus had serum concentrations of 25-hydroxyvitamin D significantly lower (*P* = 0.045) than those with chronic pancreatitis having diabetes mellitus, whereas smokers with pancreatic ductal adenocarcinoma had serum concentrations of calcium significantly higher (*P* < 0.001) as compared to nonsmokers. Altered bone metabolism seems to be associated with chronic diseases of the pancreas; however, the mechanism should be better elucidated.

## INTRODUCTION

Vitamin D together with vitamins A, E, and K are liposoluble substances and require pancreatic enzymes to be absorbed by the intestinal tract^1^; vitamin D exerts broad-ranging effects on muscle and bone calcium handling, differentiation, and development; it also modulates muscle and bone-derived hormones, potentially facilitating cross-talk between these tissues.^2^ In a clinical setting, vitamin D deficiency results in generalized atrophy of muscle and bone, suggesting coordinated effects of vitamin D at these sites.^2^ Parathormone (PHT) is a hormone secreted by the parathyroid cells^3,4^; it elevates the blood calcium level by dissolving the salts in bone and preventing their renal excretion.^5^ Osteocalcin is the major noncollagenous bone protein and is regarded as a specific index of bone formation.^6^ Bone alkaline phosphatase is a measure of bone formation.^7^ Measuring the biochemical markers of bone metabolism could lead to a better understanding of the transition of bone in chronic diseases of the pancreas; in fact, the isolation and characterization of both the cellular and the extracellular components of the skeletal matrix results in the development of molecular markers, which are considered to reflect either bone formation or bone resorption.^8^ In addition, these biochemical indices are noninvasive, have a low cost, and may be obtained routinely^9,10^; they are helpful tools in the diagnostic and therapeutic assessment of metabolic bone disease.^8^ There are no studies comparing some of the most important markers, such as vitamin D, PTH, osteocalcin, bone alkaline phosphatase, and calcium, in patients with chronic benign and malignant pancreatic diseases. Thus, the aim of our study was to comparatively evaluate serum markers of bone metabolism in patients with chronic pancreatitis and in those with ductal pancreatic adenocarcinoma.

## SUBJECTS AND METHODS

From January 2014 to December 2015, 63 consecutive subjects were studied: 30 patients with a firm diagnosis of chronic pancreatitis (based on clinical history characterized by recurrent pain associated with imaging compatible with features of chronic pancreatitis associated or not with the presence of exocrine pancreatic insufficiency), and 33 having histologically confirmed pancreatic adenocarcinoma.

Ten patients with established diagnosis of osteoporosis/osteopenia, those treated with drugs for osteoporosis/osteopenia, those with known hypogonadism, and those with renal or hepatic insufficiency, were excluded from the study.

The clinical characteristics of the patients studied are reported in Table 1. An alcohol drinker was defined when a subject actively consumes >80 g of pure alcohol per day for at least 5 years, and a smoker as a subject smoking any quantity of tobacco for at least 5 years; a patient was classified having pain when a typical pancreatic pain was present during the last 7 days before his entry into the study. From all patients, the blood samples were taken at the time of diagnosis, after informed consent was obtained. The study was approved by the Department of Digestive System of Sant’Orsola-Malpighi Hospital, Bologna, Italy, and the examinations performed are those routinely performed in these patients.

**TABLE 1:**
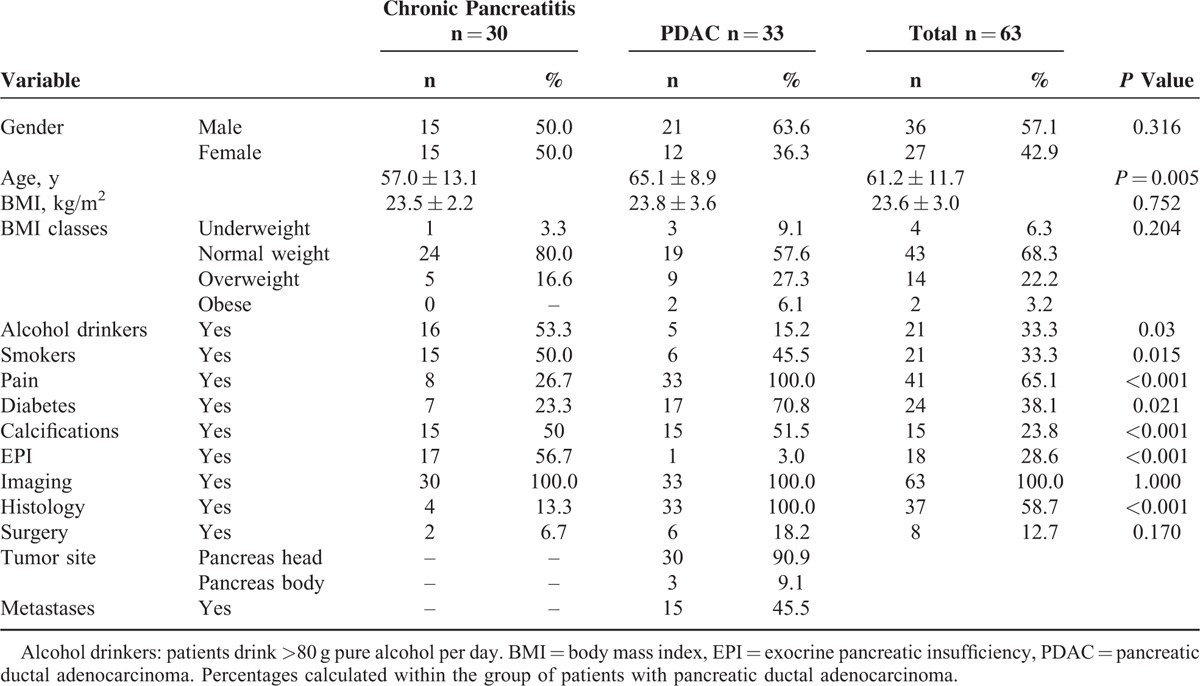
Clinical Characteristics of the 63 Patients Studied

Serum 25-hydroxyvitamin D levels were measured using a chemiluminescence assay (25 OH Vitamin D TOTAL Assay, DiaSorin, Saluggia, Italy). The within-run coefficient of variation (CV) was 3.7 to 7.7% and the total imprecision CV was 5.8 to 10.9%; the detection limit of the assay was 4 ng/mL. For the quantitative determination of bone alkaline phosphatase, a direct, 2-site sandwich-type immunoluminometric assay was utilized using 2 monoclonal antibodies (BAP OSTASE, DiaSorin, Saluggia, Italy). The within-run coefficient of variation was 3.2 to 4.0% and the total imprecision CV was 6.5 to 8.1%; the detection limit of the assay was 1.5 μg/L. Osteocalcin was assayed using a direct, 2-site, sandwich-type immunoluminometric assay with directly coated magnetic microparticles (Osteocalcin, DiaSorin, Saluggia, Italy). The within-run coefficient of variation was 3.0 to 8.0% and the total imprecision CV was 4.0 to 9.0%; the detection limit of the assay was 0.5 ng/mL. For the quantitative determination of 1 to 84 PTH without a cross-reaction to 7 to 84 and other PTH fragments, a direct, 2-site, sandwich-type immunoluminometric assay utilizing directly coated magnetic microparticle was used (1–84 PTH Assay, DiaSorin, Saluggia, Italy). The within-run coefficient of variation was 3.0 to 5.9% and the total imprecision CV was 5.5 to 9.0%; the detection limit of the assay was 4 pg/mL. All these assays were carried out using the LIAISON Analyzer (DiaSorin, Saluggia, Italy). Calcium was determined using a colorimetric method (VITROS Chemistry Products Ca Slides, Ortho Clinical Diagnostics, Rochester). The within-run coefficient of variation was 0.04 to 0.12% and the total imprecision CV was 0.9 to 1.9%.; the detection limit of the assay was 4 mg/dL. The test runs were carried out using Vitros 5600 (VITROS 5600 Integrated System, Ortho Clinical Diagnostics, Rochester).

The reference values of the various substances evaluated were as follows: 25-hydroxyvitamin D: <20.0 ng/mL was adopted as “deficient”, between 20.0 and 30.0 ng/mL as “insufficient” whereas optimal levels were defined as vitamin D >30.0 ng/mL; osteocalcin in men 4.6 to 65.4 ng/mL and in women 6.5 to 42.3 ng/mL during the premenopausal age and 6.5 to 59.1 ng/mL during the postmenopausal age, PTH: 4.6 to 58.1 pg/mL, bone alkaline phosphatase 5.5 to 24.6 μg/L and calcium 8.4 to 10.2 mg/dL. Finally, fecal pancreatic elastase-1, as a marker of exocrine pancreatic insufficiency (ScheBo Biotech AG, Giessen, Germany, the within-run CV 6.4%, the total imprecision CV 8.8%, and the detection limit 15 μg/g); values <200 μg/g were considered to be the index of exocrine pancreatic insufficiency.

### Statistical Analysis

Serum 25-hydroxyvitamin D levels and calcium concentration were normally distributed in our study population; on the other hand, osteocalcin, bone alkaline phosphatases, and PTH were not normally distributed, and their values were normalized using lognormal transformation. Thus, for the continuous variables, a parametric test (1-way ANOVA) was applied to analyze the data. For the categorical variables, the Fisher exact test and the Pearson chi-square were considered as appropriate. Finally, categorical variables such as alcohol, smoking pain, diabetes mellitus, and exocrine pancreatic insufficiency were considered as binary variables for the statistics.

## RESULTS

As reported in Table 1, the 2 groups of subjects studied were comparable for gender (*P* = 0.316) whereas, as expected, the age of the chronic pancreatitis patients was significantly lower than that of patients having pancreatic ductal adenocarcinoma (*P* = 0.005). Exocrine pancreatic insufficiency (fecal elastase 1 <200 μg/g) was present in 17 patients with chronic pancreatitis and in 1 patient with pancreatic adenocarcinoma (*P*<0.001); in particular, fecal elastase 1 concentration <100 μg/g were present in 5 chronic pancreatitis patients (16.7%). The body mass index (BMI) was similar in the 2 groups of patients (0.752). According to the WHO classification,^11^ no differences among the various classes of BMI were observed between the 2 groups of patients (*P* = 0.204). The imaging studies were compatible with chronic pancreatitis in all patients with chronic pancreatitis as well as in those with pancreatic ductal adenocarcinoma. Histology was carried out in 4 patients with chronic pancreatitis and in all patients with ductal pancreatic adenocarcinoma; 8 patients underwent surgery (2 having chronic pancreatitis and 6 having a pancreatic ductal carcinoma). It should be noted that the 2 patients with chronic pancreatitis had derivative surgery, whereas the 6 patients with pancreatic cancer had resective surgery.

Taking into consideration the clinical variables of all 63 patients studied (Table 2), 25-hydroxyvitamin D was inversely correlated with only the BMI (*P* = 0.007), whereas it was not correlated with age (*P* = 0.583) and fecal elastase-1 concentrations (*P* = 0.556). Regarding the other substances studied, PTH was positively correlated with only the age of the patients (*P* = 0.015).

**TABLE 2:**
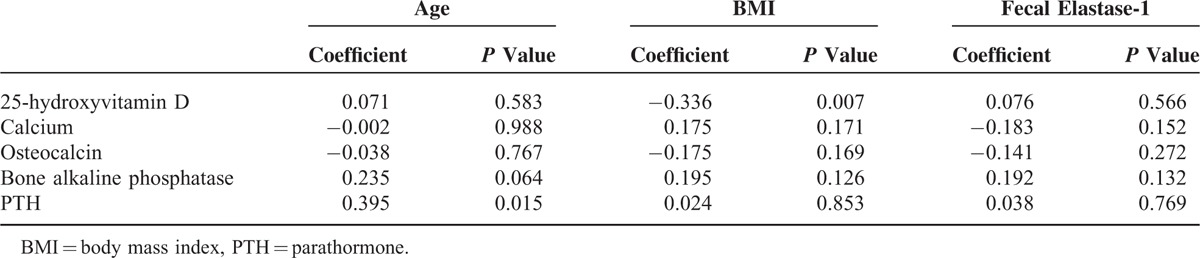
Correlation Between Age, BMI, and Fecal Elastase-1 Concentrations, and the Substances Studied

As reported in Table 3, of the 5 substances studied, only bone alkaline phosphates were significantly different (*P* < 0.001) between patients with chronic pancreatitis (mean ± SD; 12.2 ± 1.7 μg/mL) and those with pancreatic ductal adenocarcinoma (36.6 ± 2.0 μg/mL).

**TABLE 3:**
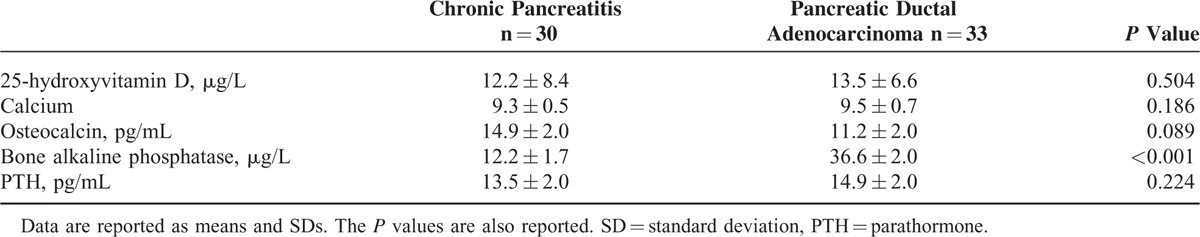
Serum Concentrations of the 5 Substances Studied in Patients With Chronic Pancreatitis and in Those With Pancreatic Ductal Adenocarcinoma

The 5 substances were also not related to gender, alcohol habit, smoking habit, presence of pain, presence of exocrine pancreatic insufficiency, presence of diabetes mellitus, or surgery (Table 4). Within the 2 groups of patients, the 23 patients with chronic pancreatitis without diabetes mellitus had serum concentrations of 25-hydroxyvitamin D (11.7 ± 7.1 ng/mL) significantly lower (*P* = 0.045) than those with chronic pancreatitis and diabetes mellitus (13.9 ± 12.3 ng/mL) whereas smokers with pancreatic ductal adenocarcinoma had serum concentrations of calcium (9.9 ± 1.3 mg/dL) significantly higher (*P* < 0.001) as compared to nonsmokers (9.4 ± 0.5 mg/dL). Within the group of patients with pancreatic ductal adenocarcinoma, there were no differences in serum levels of the 5 markers studied between the 18 patients without metastases and the 15 with metastases (Table 5). No differences in the 5 markers studied were also found between patients with local cancer invasion and those with distant metastases.

**TABLE 4:**
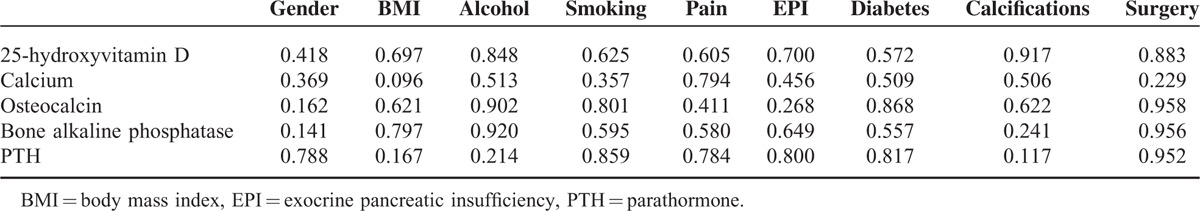
Significance of the Relationship Between the 5 Substances and Gender, Classes of Body Mass Index (BMI), Alcohol Habit, Smoking Habit, Pain, Exocrine Pancreatic Insufficiency, and Diabetes

**TABLE 5:**
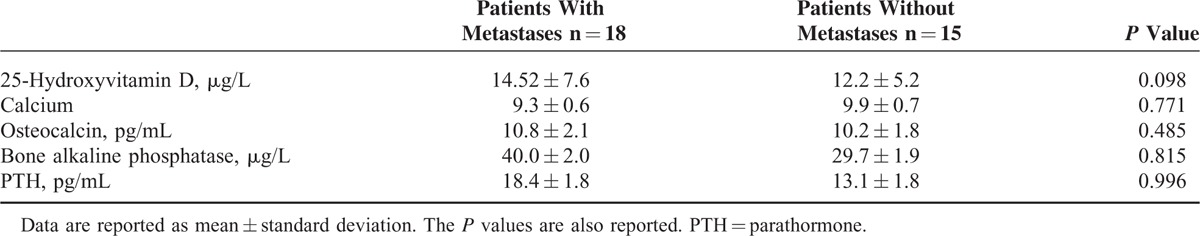
Serum Concentrations of the 5 Substances Studied in Patients with Pancreatic Ductal Adenocarcinoma With and Without Metastases

**FIGURE 1:**
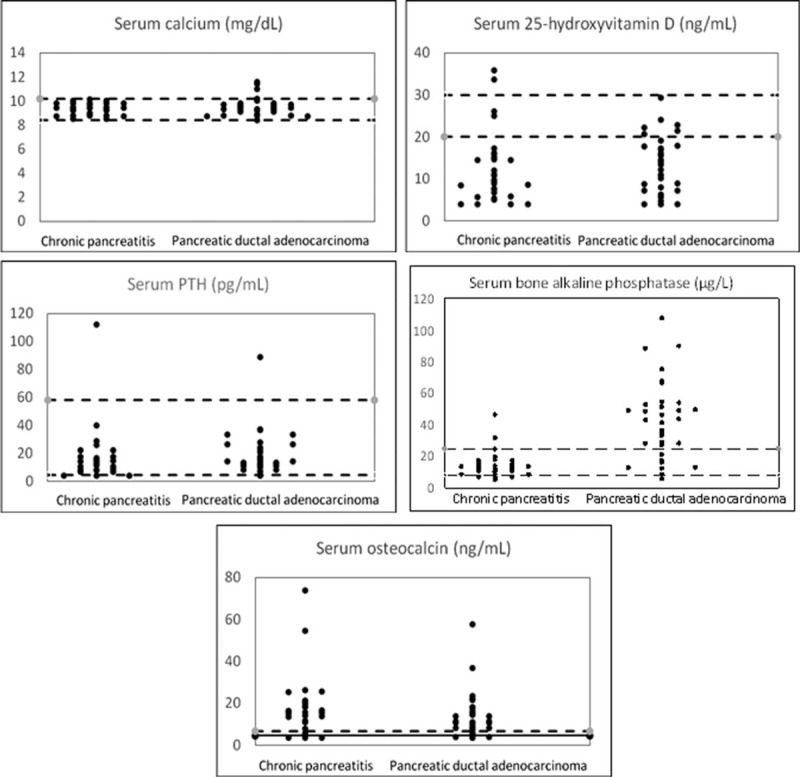
Individual values of the 5 substances studied in patients with chronic pancreatitis and in those with pancreatic ductal adenocarcinoma. The horizontal dashed lines represent the limits of 25-hydroxyvitamin D: “deficient” (<20.0 ng/mL), “insufficient” (between 20.0 and 30.0 ng/mL) and optimal levels (>30.0 ng/mL); the horizontal dashed lines of calcium, parathormone (PTH), and bone alkaline phosphatase represent the upper and low reference limits; the horizontal solid line of osteocalcin represents the lower normal limit in men and the horizontal dashed line, the lower normal limit in women. PTH = parathormone.

Finally, the frequencies of the abnormal values of the 5 substances evaluated in the 2 groups of patients studied are reported in Figure 1. In particular, 26 patients with chronic pancreatitis (86.7%) had deficient levels of 25-hydroxyvitamin D (2, 6.7% had insufficient serum concentrations of this vitamin), 2 (6.7%) had high serum concentrations of this vitamin, and only 2 had normal concentration (6.7%). Twenty-seven patients with pancreatic ductal adenocarcinoma (81.8%) had deficient levels of 25-hydroxyvitamin D, 6 (18.2%) had insufficient levels and none had normal levels. Four patients with chronic pancreatitis (13.3%) and 6 patients with pancreatic ductal adenocarcinoma (18.2%) had low levels of osteocalcin. Two patients with chronic pancreatitis (6.7%) had high serum levels of bone alkaline phosphatases, whereas 25 patients with pancreatic ductal adenocarcinoma (75.8%) had elevated levels of this protein; this difference was statistically significant (*P* < 0.001). Calcium was normal in all patients with chronic pancreatitis and was abnormally high in 3 patients with pancreatic ductal adenocarcinoma (9.1%). In the chronic pancreatitis group, 3 patients had low levels of PTH, 1 (3.3%) had elevated levels of this hormone, and 26 patients had normal levels (86.7%); these figures were similar to those found in patients with pancreatic ductal adenocarcinoma (1 patient had low levels, 3.0%, 1 had high levels, 3.0%, and 31 had normal concentrations, 93.9%).

## DISCUSSION

Improvement of nutritional status significantly decreased risk of mortality independent of sex, previous treatment history, and evidence of biological anticancer activity.^12^ This aspect should be taken in consideration in patients with pancreatic cancer and the medical therapy of bone metabolism is a part of the nutritional support.

Data regarding vitamin D deficiency in chronic pancreatitis diseases are still under debate. It is well known that both deficiency of fat-soluble vitamins and decreased bone mineral density are frequently present in chronic pancreatitis patients with and without exocrine insufficiency, suggesting that routine screening to detect altered bone metabolism should be carried out.^13–15^

Our data confirmed that >90% of chronic pancreatitis patients had deficient or insufficient serum concentrations of 25-hydroxyvitamin D; it was also found that ∼13% of them also had low levels of osteocalcin, 7% had high serum levels of bone alkaline phosphatases, whereas calcium was normal in all chronic pancreatitis patients. These results showed that only a low percentage of chronic pancreatitis patients had a severe altered bone metabolism. Considering the clinical variables associated with the 5 substances studied, it was found that only 23 patients with chronic pancreatitis without diabetes mellitus had serum concentrations of 25-hydroxyvitamin D significantly lower than those with chronic pancreatitis and diabetes mellitus. The factors related to an altered bone metabolism are still under debate. Some authors have reported that low fecal elastase-1 correlated with low bone mineral density in conventional x-rays (*P* < 0.05) in chronic pancreatitis patients, and that patients receiving pancreatic enzyme replacement therapy had significantly higher dual-energy x-ray absorption values^16^; the same results have been obtained by others.^17^ However, the results of a meta-analysis have shown that factors other than pancreatic insufficiency are related to the high risk of osteoporosis in chronic pancreatitis patients,^18^ and our data confirmed these results. It has been reported that altered bone turnover in chronic pancreatitis is associated with both smoking, and systematic inflammation was identified.^19^ Moreover, our results regarding the smoking habit and low levels of vitamin D seems to be different and we need of more studies on this topic.

Regarding patients affected by pancreatic ductal adenocarcinoma, it was also found that ∼82% of these subjects had deficient levels of 25-hydroxyvitamin D and 18% had insufficient levels. Approximately 20% had low levels of osteocalcin and 76% had elevated levels of alkaline phosphatase; this latter figure was significantly higher as compared to chronic pancreatitis patients. Finally, calcium was abnormally high in ∼9% of the patients with pancreatic ductal adenocarcinoma and these patients could have a paraneoplastic syndrome.^20^ In pancreatic cancer patients, the results of a systemic review have shown that dietary vitamin D or circulating concentrations of 25-hydroxyvitamin D are not associated with the risk of pancreatic cancer.^21^ Other authors have found that, in men, there is an increased risk of pancreatic cancer associated with currently recommended dietary vitamin D intake levels; no associations with vitamin D intake were observed among women.^22^ On the contrary, a genetic study found that 2 high-risk genotypes (ie ffbb and Ffbb) had an 11.66 and 6.42-fold increased risk of pancreatic cancer, respectively; furthermore, vitamin D receptor gene polymorphisms were important in the development of pancreatic cancer.^23^ Others^24^ have also confirmed these data suggesting that some variants in vitamin D-related genes may influence pancreatic cancer risk. Several cancers have shown an increased risk associated with low vitamin D levels, suggesting that vitamin D supplementation may be an optimal strategy to reduce the risk of different malignancies. On the other hand, it has also been reported^25^ that high vitamin D levels may be associated with an increased risk of pancreatic; in fact, high vitamin D binding protein concentrations may sequester more 25(OH)D and reduce free 25(OH)D bioavailability. It is possible that, in our patients with pancreatic cancer, bone metabolism was altered and this alteration was probably due to the reduced alimentary introduction of vitamin D and calcium; this was supported by the fact that we found an inverse relationship between vitamin D and the body mass index.

One bias of this study is that we have not evaluated both serum C-terminal cross-linking telopeptide of type I collagen (CTX-1) as a marker of bone resorption and serum procollagen type I N propeptide (s-PINP) as a marker of bone formation^26^; however, we have used only osteocalcin and, although this is appropriate, we believe that because bone metabolism is a coupled process, measurement of both CTX-1 and s-PINP is important to better understand the world of bone metabolism in patients with chronic diseases of the pancreas and we believe that this aspect requires further study.

In conclusion, an altered bone metabolism seems to be associated with chronic diseases of the pancreas, but the mechanism should be better elucidated because low serum levels are not associated with gender, exocrine pancreatic insufficiency, alcohol and smoking habits, pancreatic calcifications, pain, and diabetes mellitus. PTH was positively correlated with the age of our patients and 25-hydroxyvitamin D was inversely correlated with only the BMI.
